# Progressive Vertical and Horizontal Phytocompound Changes during Agarwood Formation in *Aquilaria sinensis* after *Geotrichum candidum* Injection

**DOI:** 10.3390/life13112147

**Published:** 2023-10-31

**Authors:** Lih-Geeng Chen, Amalia Dyah Arumsari, Chishih Chu

**Affiliations:** 1 Department of Microbiology, Immunology and Biopharmaceuticals, College of Life Sciences, National Chiayi University, Chiayi City 60004, Taiwan; lgchen@mail.ncyu.edu.tw; 2 Global Master Program of Life Sciences, College of Life Sciences, National Chiayi University, Chiayi City 60004, Taiwan

**Keywords:** *Aquilaria sinensis*, *Geotrichum candidum*, agarwood, phytocompounds, biochemical analysis

## Abstract

(1) Background: Agarwood is an aromatic resin produced by the host tree through an immunological response against biotic and abiotic stress. The aim was, first, to use the fungus *Geotrichum candidum* to stimulate compound changes in *Aquilaria sinensis* horizontally (color formation) and vertically (cutting layers) after injection with it. (2) Methods: Horizontal and vertical sections were collected and separated five months after injection with the fungal broth. Two grams of dry powder was mixed with 20 mL methanol for 3 h at room temperature, and the solution was vibrated in an ultrasonic cleaner bath at 40 °C for 1 h. After vacuum drying, a concentration of 10 mg/mL of the tested samples in methanol was prepared for reversed-phase high-performance liquid chromatography (RP-HPLC), gas chromatography/mass spectrometry (GC/MS), and thin-layer chromatography (TLC) analysis. (3) Results: The horizontal changes in the compounds and their concentrations were associated with color. Compared to the normal (N) group, *G. candidum* injection stimulated more compounds at RT 27–42 in the white (W) group, brown (BR) group, and black (B) group. Furthermore, a significant increase in fatty acids was observed in the W group, implying an early plant response after *G. candidum* injection. In the BR group, the compounds were more similar to commercial agarwood (Out group). In the B group, alkaloids were the main compounds. Vertical changes in the main compounds were not observed, although the compound level varied. A TLC analysis determined the main compounds in the BR group at 254 nm and in the B group at 365 nm. Higher fatty acid levels were found in L6 and L5 and were correlated with higher terpenoid and sesquiterpene levels, suggesting that these compounds were possibly the first stage of agarwood formation. A GC/MS analysis demonstrated that the main compound groups were almost identical to the BR parts. (4) Conclusions: The injection of *G. candidum* led *A. sinensis* to synthesize different phytochemicals horizontally, not vertically, in the BR group.

## 1. Introduction

Agarwood is a blackish resinous heartwood on the trunk and branches of the entire tree [1], mainly from the genera *Aquilaria* and *Gyrinops* [2,3,4,5,6]. Agarwood accounts for 7–10% of the resin in *Aquilaria* trees [7]. Agarwood is widely used for incense, perfume, and medicine [7,8]. Under the Convention on International Trade in Endangered Species of Wild Fauna and Flora (CITES), *Aquilaria* has been protected as an endangered species since 2004. Lacking natural wild agarwood stocks, artificial agarwood has been produced through wounding processes using biotic and abiotic stresses [9]. These stresses activate the immune responses of plants to synthesize secondary metabolites through several pathways, including the shikimic acid pathway to synthesize aromatic amino acids, particularly l-phenylalanine, l-tyrosine, and l-tryptophan as precursors for alkaloids and phenolics; the malonic acid pathway to synthesize fatty acids and polyketides; and the mevalonate pathway in the cytosol, or the 2-methylerythritol 4-phosphate pathway in the plastids, to synthesize five-carbon (C5) building blocks, isopentenyl pyrophosphate (IPP), and dimethylallyl pyrophosphate (DMAPP).

The important phytocompound groups in plants are terpenes/terpenoids, phenolic compounds, and nitrogenous compounds. With C5 isoprene units, terpenoids are derived from terpenes with elements other than carbon (C) and hydrogen (H) molecules, such as oxygen (O), after terpene oxidation. One of the terpenes, sesquiterpene (C15), has been reported to have several medical functions, such as regulation for the prevention of cancer growth [10]. Phenolics from shikimic acid and the phenylpropanoid pathways differ in their bioactive properties depending on their functional groups. The bioactive nitrogenous compounds are tryptamine derivatives, purine alkaloids, and amide derivatives. In plants, alkaloids are small organic molecules that form a ring with nitrogen and account for approximately 20% of the compounds that defend plants against pathogens [11,12].

The important factors for agarwood formation include the fungal invasion of *Aquilaria* trees [13], mechanical injuries to crack the bark, and insect attacks to form cracks and crevices along the bark for microbial invasion and colonization [7]. *Geotrichum candidum* can invade several plants, such as soybean, strawberry, tomato, and peach, under favorable environmental conditions. *G. candidum* can be disseminated by both wind and water, and invasion can occur in wounds and under stress environments [14]. However, this fungus has not been tested to stimulate agarwood formation in *A. sinensis*.

In *A. sinensis*, the agarwood properties are significantly related to the presence of terpenes, such as monoterpenes, diterpenes, and sesquiterpenes, which are closely related to the fungal species [15]. Furthermore, the levels of volatile components and alcohol extract contents can be reduced under a relatively low moisture content [16]. To better understand agarwood formation in *A. sinensis* after *G. candidum* injection, we characterized the phytocompound differences among the horizontal color zones in a trunk layer and among the vertically cut layers in an upper branch of the injection site.

## 2. Materials and Methods

### 2.1. Sample Preparation

**Figure 1:**
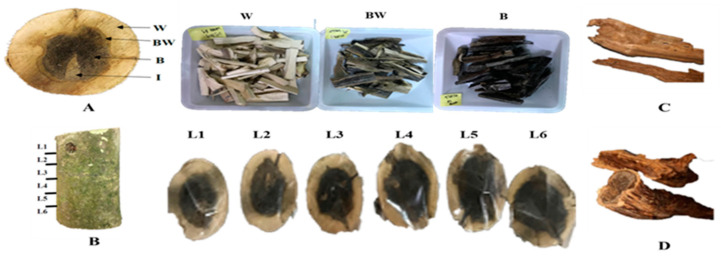
Agarwood from *A. sinensis* after *G. candidum* injection. (**A**) Trunk samples: W—white part; BW—brown part; B—black part; I—site above the injection. (**B**) Vertical layers of an upper branch: L1–L6: layer 1–layer 6. (**C**) Commercial agarwood. (**D**) Previous agarwood produced from the same plantation.

The *A. sinensis* plantation is located in Nantou, Taiwan. A mature tree trunk with a diameter of 24 cm was injected with *G. candidum* (accession number OR077470), and the trunk was collected five months after injection (Figure 1A). The trunk was separated into white (W), brown (BR), and black (B) parts. An uninjected trunk was used as the control (N). The branch above the injection site was cut off vertically into six layers. Each layer was four centimeters apart and separated horizontally into three color parts (Figure 1B). Furthermore, dry agarwood samples were obtained from a commercial market (Out group, Figure 1C) and the plantation (In group, Figure 1D).

### 2.2. Methanol Extraction of Polar Compounds

Agarwood preparation and extraction were carried out by modified methods [17,18]. Each agarwood sample was dried in an oven at 40 °C for 48 h, and then dry agarwood was ground to powder of an appropriate size. Two grams of agarwood powder was immersed in 20 mL of 100% methanol for 3 h at room temperature, and the solution was treated in a DELTA^®^ DC200H ultrasonic cleaner bath at 40 °C for 1 h. The extractions were dried in a Buchi rotary evaporator (Buchi, Saint Gallen, Switzerland) at 40 °C with a PANCHUM VC-7600 vacuum controller at 700 mmHg for 15 min until methanol was fully evaporated. The dried extract was dissolved in methanol to prepare a concentration of 10 mg/mL for subsequent analysis.

### 2.3. Thin-Layer Chromatography (TLC) Analysis of Vertical Layers

The compounds in three color parts from six vertical layers were analyzed by TLC analysis. The 18 samples were spotted on a silica gel 60G F254 TLC plate (Merck, Rahway, NJ, USA) and then placed in a capped chamber containing the mobile phase hexane/acetone with an 8:2 ratio. The plates were dried using a rotary evaporation BUCHI Heating Bath B-490 at 40 °C with a PANCHUM VC-7600 vacuum controller (Panchum Scientic Corp, Taipei, Taiwan) until the N-hexane and acetone solvent was fully evaporated. The compounds were detected with UV detectors at wavelengths of 254 nm and 365 nm.

### 2.4. Reversed-Phase High-Performance Liquid Chromatography (Rp-Hplc) Analysis

RP-HPLC analysis was performed using a LiChrospher 100 RP-18e (4 mm i.d. × 250 mm, 5 μm) (Merck, Rahway, NJ, USA) with mobile phases A (0.05% trifluoroacetic acid in water) and B (CH3CN) at a column temperature of 40 °C. A 10 μL sample was injected at a flow rate of 1 mL/min. The wavelengths of 230 nm and 280 nm were used to detect mostly the saponin groups in the plant compounds [19], the compounds without a good chromophoric group [20], and mostly phenolic groups [21]. The linear gradient ratios of 0.05% trifluoroacetic acid–CH3CN were as follows: 0–10 min, 90:10; 10–15 min, 85:15; 16–30 min, 70:30; 31–55 min, 10:90; 55–56 min, 90:10; 57–66 min, 90:10.

### 2.5. Gas Chromatography/Mass Spectrometry (Gc/Ms) Analysis

GC/MS analysis was carried out using GC-2010 with GCMS-QP 2010 Shimadzu (Shimadzu, Kyoto, Japan) with a fused silica DB-5MS capillary column (30 cm × 0.25 mm × 0.25 µm film thickness). First, the column was set at 70 °C for 2 min, followed by an increase to 200 °C at a rate of 15 °C/min and held for 5 min, an increase to 250 °C at a rate of 20 °C/min and held for 3 min, and an increase to 320 °C at a rate of 20 °C/min and held for 5 min. Helium was used as the carrier gas with a constant flow rate of 1.15 mL per minute, and the ion-source temperature was set at 200 °C. The chemical compounds were identified by comparing the sample mass spectrum to the National Institute of Standards and Technology (NIST) mass spectral library (NIST147. LIB; NIST27. LIB; NIST17s. LIB). Compounds with lower match probabilities (less than 75) were regarded as unknowns. The chemical compounds are presented in terms of abundances (%) by the peak area normalization method.

## 3. Results

### 3.1. RP-HPLC Analysis of Compound Changes during Agarwood Formation

#### 3.1.1. Compound Changes among Three Color Parts Horizontally 

**Figure 2:**
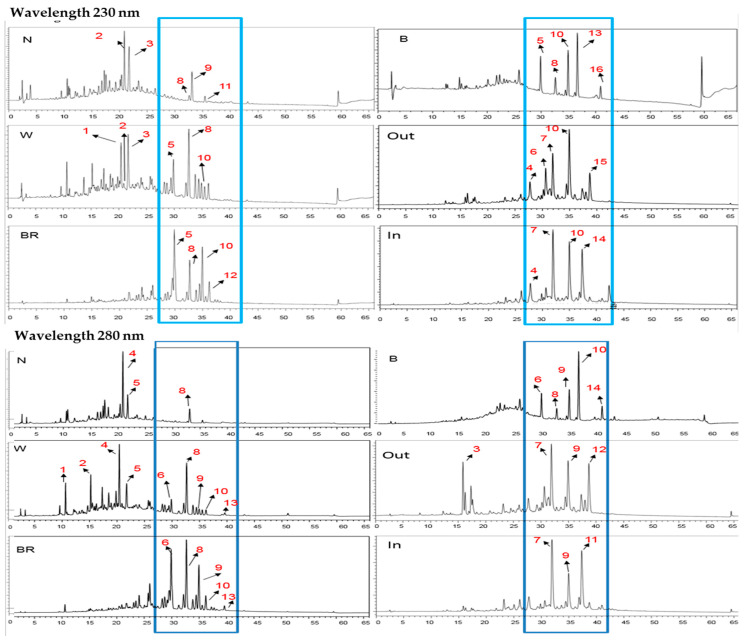
RP-HPLC chromatogram of the main compounds in different color parts horizontally detected at wavelengths of 230 nm and 280 nm. N: non-injected wood, W: white part, BR: brown part, B: black part, Out: agarwood from market, and In: agarwood from same plantation. Number indicates the major bands identified at both wavelengths, separately. Blue box indicates main phytocompounds presents in all parts, nearly missing in the N group.

*G. candidum* injection changed the phytocompounds among three color parts and the control (N) group. At 230 nm, 16 main compounds were separated into two ranges with RT10–25 in the N and W groups and RT27–42 in the W, BR, and B groups of fresh wood and agarwood groups (Figure 2). Three common compounds in all groups displayed different patterns: peak 3 (RT21.69), with similar concentrations in the N, W, and BR groups and lower concentrations in the B, Out, and In groups; peak 5 (RT29.87), with an increase from the N group to the W and BR groups and a reduction to the B, Out, and In groups; and peak 12 (RT36.18), with an increase from the N group to the W and BR groups and the lowest concentrations found in the B group (Table 1). Furthermore, peaks 10 (RT34.93) and 13 (RT 36.55) were present in all groups, except the N group. Two compounds (peaks 5 and 8) appeared in all fresh wood groups, while peaks 4 and 7 were commonly found in both agarwood groups. Nevertheless, peaks 1, 5, 8, and 10 increased from the N group to W group and BR group possibly due to the molecules responsible for early response to *G. candidum* injection. Furthermore, the B group contained the lowest compound number and concentration, while the BR group had the highest compound content.

Under a wavelength of 280 nm, usually for flavonoid compounds, 14 main compounds were separated into two main ranges identical to those of 230 nm with RT10–25 in the N and W groups and with RT27–42 in the W, BR, and B groups of the fresh wood and two agarwood groups. Common compounds were observed for peak 8 in all fresh wood groups and Out group; peak 9 in the W, BR, and B groups and two dry agarwood groups; peaks 6, 8, 9, and 10 only in the W, BR, and B groups; and peaks 7 and 9 in the two agarwood groups, suggesting that peak 9 can be used as an agarwood marker. Nevertheless, compounds at peaks 11 and 12 differed between the two agarwood groups. Compounds at peaks 1, 2, 6, 9, and 10 differed between the N group and W group, implying that they may be the molecules seen in *G. candidum* injection. Seven phenolics appeared to have identical RTs between the two wavelengths at RT20.9, RT21.7, RT29.9, RT34.9, RT36.2, RT38.7, and RT40.9. However, only compounds at RT21.7, RT29.9, and RT36.2 were present in all groups, and the remaining compounds displayed different levels among groups (Table 1). Furthermore, BR had the highest compound number and levels, and two compounds at RT32.1 and RT34.9 were highest in the BR, Out, and In groups and lacking in the N group, implying that these two compounds may be markers for agarwood induced by *G. candidum*.

#### 3.1.2. The Compound Changes Vertically among the Three Color Parts in the Upper Branch

Several factors may cause phytochemical changes in agarwood formation and sample preparation and storage. Compared to the main phytocompounds in the trunk horizontally, phytocompounds were detected more at 230 nm than at 280 nm and, conversely, phytocompounds in the upper branch were detected more at 280 nm than at 230 nm. Furthermore, the phytocompound number differed among the three color parts, but was almost identical among the six vertical layers with various concentrations (Table 2). Common phenolics were identical at RT20.9, RT21.7, RT29.9, RT34.9, and RT36.2 between two wavelengths in the trunk and in the vertical layers, but these compounds displayed different patterns among the tricolor parts.

Common compounds in all groups were observed at RT29.9, RT32.6, and RT34.9 detected at 230 nm and at RT20.4, RT25.7, RT29.9, and RT32.6 detected at 280 nm. At 230 nm, the main compounds with a weight ratio larger than >3% were at RT21.7 and RT32.6 for the W group and at RT29.9, RT32.6, and RT34.9 for the BR and B groups. Color-specific compounds were found only in the W and B groups. At 280 nm, eleven color-specific compounds were found in the W group, three in the BR group, and six in the B group. The main compounds with a weight ratio larger than >2% were RT21.7, RT29.9, and RT32.6 for the W group; RT26.0, RT29.4, RT29.9, RT32.1, RT32.6, RT34.8, and RT36.2 for the BR group; and RT21.7, RT22.3, RT26.0, RT29.9, RT32.6, RT34.9, and RT36.6 for the B group.

In the combination of phytocompounds detected at both wavelengths, the ratio between 230 nm and 280 nm was different between fresh wood samples (4:1) and agarwood samples (1:5.5 for the Out group and 1 to 2.2 for the In group) (Table 3). Using the area in the N group as control, there was an increase in the W and BR groups and a reduction in the B and Out and In groups at 230 nm, while a different pattern was observed at 280 nm with a ratio of 1.9 for the W and BR groups, larger than 8 for the Out and In groups, but 0.23 for the B group. These results suggest a dramatic increase in the compound area, especially in both agarwood samples, at 280 nm and that differed in phytocompounds between agarwood and fresh wood samples. Further, in a vertical comparison of the area in the BR group with L6 as control, no sequential order was observed with a ratio larger than 1 for the L5 and L2 layers and smaller than 1 for L3 and L1 layers. 

### 3.2. TLC Analysis of the Compound Changes among Three Color Parts Vertically

**Figure 3:**
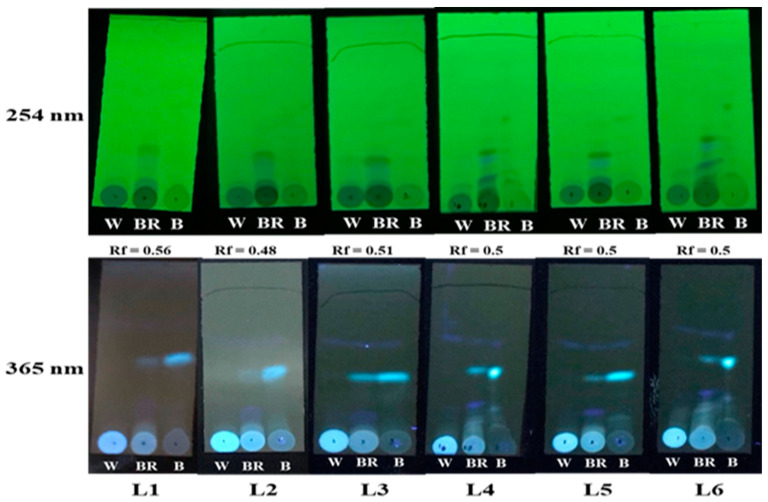
Thin-layer chromatography (TLC) analysis of phytocompounds in the W, BR, and B groups from six vertical layers vertically detected at wavelengths of 254 nm and 365 nm.

Thin-layer chromatography (TLC) analysis provides qualitative information about the phytocompound groups in the plant extract and screens rapidly the differences in phytocompound groups among tricolor parts before RP-HPLC analysis. At a wavelength of 254 nm, phytocompounds were rich in the BR group and then almost absent in the B and W groups. On the contrary, at a wavelength of 365 nm, two blue color bands were observed with one in all groups and one only present in the BR group from layers L2 to L6; white color bands were observed in the B group, fewer in the BR group, and absent in the W group (Figure 3).

### 3.3. GC/MS Analysis

#### 3.3.1. Changes in Compound Groups

**Figure 4:**
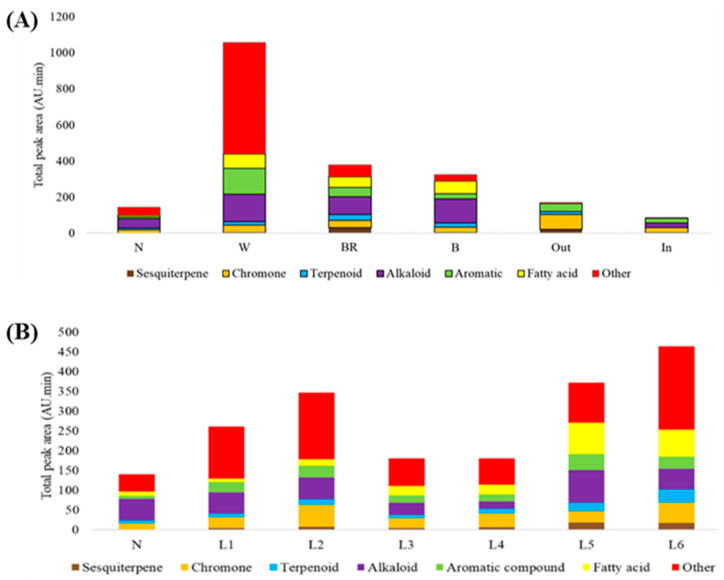
GC/MS analysis of the differences in compound groups horizontally (**A**) and vertically (**B**).

Color change represents complex processes of compound synthesis and accumulation through different pathways during microbial invasion and during tree aging. GC/MS analysis separated compounds into six known compound groups that differed in total levels among fresh wood groups and agarwood groups and displayed higher compound levels in the trunk than in the vertical parts of the upper branch (Figure 4, Tables S1 and S2). The agarwood groups displayed the lowest compound levels and lacked compounds in the other group that appeared in all fresh wood groups; fatty acids and aromatic compounds were the two main compounds (Figure 4A). In the fresh wood groups, total phytocompound levels were highest in the W group with the highest levels of other compounds, followed by the BR group, B group, and N group. Compared to the compound groups in the N group, *G. candidum* injection stimulated more compounds in all compound groups, especially other compounds for the W group. Sesquiterpenes were detected only in the BR and Out groups, implying that these compounds may be important for agarwood formation after *G. candidum* injection. Vertical compound comparison displayed two patterns with the highest levels in L6 down to L3 and an increase from L3 to L2 and a decrease from L2 to L1 (Figure 4B). Apart from other compounds, the most abundant compound groups were fatty acid and aromatic compounds in L5 and L6, chromones in L6 and L2, alkanoids in L5, and terpenoids in L6. Chromones and terpenoids are the two main compounds in agarwood. 

#### 3.3.2. Changes in Main Compounds in Different Compound Groups

Differences in the main compounds of each compound group differed among sequiterpenes, chromones, terpenoids, and alkaloids (Table 4). In general, the BR group and the B group showed common main compounds. Identical compounds among the four compound groups were found at RT13.1 for terpenoids, at RT24.73 for alkaloids, at RT10.625 for aromatic compounds, and four compounds at RT16.02, RT16.58, RT18.33, and RT18.75 for fatty acids. The highest level in each compound group was observed for compounds at RT18.75 for fatty acids and at RT17.03 for others in the W group, at RT12.37 for sesquiterpenes in the BR group, and at RT24.73 for alkaloids in the B group.

The compounds with the highest levels were alkaloids and fatty acids for the BR group and L5/L6, chromones and aromatics for L1, chromones and alkaloids in L2/L3, and chromones and fatty acids in L4 (Table 4). Alkaloid levels were especially high in L6, L5, L3, and L2, while fatty acids were high in L6 and L5. In all BR parts, the main compounds were at RT12.27, RT13.86, and RT15.74 for sequiterpenes, at RT10.9 and RT24.38 for chromones, at RT8, RT8.33, and RT 13.12 for terpenoids, at RT4.59 and RT10.56 for aromatic acids, and at RT18.34 for fatty acids. Among the six layers, the highest compound levels in each compound group were at RT12.27 and RT17.11 for sequiterpenes for L5 and L6, at RT24.02 for L3–L6 and at RT24.38 for L1–L2 and L4–L6 (>1.0) for chromones, at RT17.28 for L5–L6, at RT23.97 in L1–L2, and RT24.725 and RT35.57 in L1 (>1.0) for alkaloids, at RT4.59 and RT21.56 in L6 and at 19.29 in L5 (>0.70) for aromatic compounds, at RT16.58 and RT18.34 in L5 and L6 (>1.0) for fatty acids, and at RT16.58 in L1, RT16.63 in L4, and RT16.97 in L5 for others.

## 4. Discussion

The functions of secondary compounds change by (1) formation of a less reactive complex, (2) modification of the environmental inhibitory reactions, (3) degradation, (4) addition of functional groups, (5) conjugation which changes solubility, and (6) alteration of metabolic rate. These secondary compounds in agarwood formation can be affected by (a) species/origin of the agarwood tree, (b) methods of stimulation of agarwood resin, (c) extraction method of agarwood, and (d) tree species [22]. The microbe invasion begins at wounded tissue and then spreads along the wood ray and the cambium tangentially between the parenchymatous cells of the vascular bundles until they reach the vessel, which is correlated with wood degradation [23,24]. Vertical movement of microbes in the xylem can be affected by the amount of water because phyllosphere microbes and above-ground tissues are generally more vulnerable to water stress [25].

*A. sinensis* can synthesize phytoalexins for immune defense to induce agarwood formation mainly in the parenchyma cells of the interxylary phloem and xylem rays through fungal mobility and plant transpiration [1,26]. In early wounding, *A. sinensis* stimulates a wound-responsive gene to activate the octadecanoid pathway and jasmonate synthesis [7]. The horizontal color changes imply the progressive synthesis and accumulation of secondary metabolites, such as phenolics and terpenoids, produced through the shikimate/phenylpropanoid pathway (Figure 3 and Figure 4) [27,28,29]. In this study, peaks in RT27–42 might have a role in plant defense mechanisms and agarwood formation after *G. candidum* injection (Figure 2). Peaks 1, 5, and 10 appeared in the W group and were lacking in the N group, indicating that these compounds may be involved in early *G. candidum* invasion (Figure 1).

*A. sinensis* contains a high amount of sugar residue or several polysaccharides in the microfibrillar and amorphous phases [16]. Acid or alkali hydrolysis releases a number of soluble phenolic acids associated with lignin or simple glycosides [28]. The activity of phenolics depends on their structural diversity, such as the high antifungal activity of flavonoids, coumestrol, and glyceolin [30]. The present study demonstrated a difference in the ratio of 230/280 nm (representing changes between terpenoids and flavonoids in phenolics) between fresh wood and dry agarwood (Table 3), indicating that this change is associated with moisture content. Some studies have reported that dark-black agarwood is considered the best-quality agarwood with the highest resin content [31,32,33]. In contrast to the agarwood grade in kyara, the light-brown color of agarwood presents good quality; however, the deep color of agarwood indicates the best quality of secondary metabolites in older trees [34]. *G. candidum* invasion changed the color parts horizontally, which was associated with the difference in the main phenolics and terpenoids (Figure 3). Compared to the compounds in the N group, the compounds in the W group might result from the early-stage pathway to synthesize jasmonate, which is a general inducer of terpenoid indole biosynthesis [35]. 

Agarwood formation is recognized by the presence of a dark-brown area or discoloration zone surrounding the injected wounding sites [36]. As the main constituents in agarwood, sesquiterpenoids and terpenoids are synthesized through the mevalonic pathway, fatty acids are produced via the malonate pathway, and chromones and alkaloids are obtained from the shikimate pathway [37,38]. Volatile and hydrophobic compounds can accumulate in specific structures, such as terpenes in trichomes [7,39]. In the presence of biotic attack, free fatty acids can stimulate the synthesis of volatile compounds, such as phenolics and aromatic compounds, through oxidative burst and fatty acid oxidation cascades via the lipoxygenase pathway and lead to the production of jasmonic acid to activate the synthesis of alkaloids, terpenoids, phenylpropane, amino acid derivatives, antinutritional proteins, and some pathogen-related proteins [40]. A dramatic increase in fatty acids from the N group to the W group confirms the early stage of microbial invasion, and then the infected plants can hydrolyze starch grains to form non-starch polysaccharides with increasing phenolic substances, which may be involved in resin synthesis in agarwood [31]. In the BR group, the main compounds were the sesquiterpenes β-eudesmol and 6-benzyloxy-3,4-dihydro-4,4-dimethyl-coumarin as well as the terpenoid ingenol. The synthesis of sesquiterpenes starts with acetyl-coenzyme A in the mevalonic acid (MVA) pathway to produce terpenoids and sesquiterpenes from glycolysis [41]. However, the tree also synthesizes AsCHS1 proteins for chromone synthesis via chalcone synthase [42]. Chromones are released later after the tree releases secondary metabolites immediately after infection, and the chromone level can be increased after cell death [13] and appeared at the highest level in dry agarwood (Figure 4). Furthermore, nitrogen starvation can induce the expression of 3-deoxy-D-arabino-heptulosonate-7-phosphate synthase (DAHPS) and excitatory postsynaptic potential synthase (EPSPS) in response to a specific inhibitor of histidine biosynthesis linked to the defense response [43]. 

Microbial invasion can stimulate the continuous synthesis of agarwood-related constituents in the invasion area [44] to activate different biochemical pathways to synthesize compounds, such as chromones detectable during the first 20 days after invasion, with the main chromones becoming steady after nine months [45], suggesting complex and dynamic processes for agarwood formation after microbial infection. The vertical extent of invasion is longer than the horizontal extent [25]. The resinous substances in the epithelial cells are transported through the matrix vertically and through horizontal ducts from the pith [46]. In the BR group, the main terpenoids and phenolics differed among six layers, but showed the highest levels in the L5 layer and the lowest compound levels in the L6 layer (Figure 4), suggesting progressive changes in vertical phytochemical levels that are associated with water transpiration and fungal movement. An important difference between fresh wood and agarwood was the lack of fatty acids and alkaloids in the Out group of dry agarwood. Chromones and sesquiterpenes have a high polarity index and are easier to elute in water through transpiration [47]. Microbial invasion occurred in the lower part of the tree and spread to the entire tree, possibly through transpiration [1]. The injection of *G. candidum l*eads *A. sinensis* to synthesize secondary metabolites to suppress microbial attack and then causes necrosis of the host cells [48]. Alkaloids produced in AAA pathways act as defense compounds in plants and are efficient against pathogens and predators due to their toxicity [49]. Here, alkaloid and fatty acid levels were higher, especially in L6 and L5. These compounds are regarded as signals that trigger oxidative burst and fatty acid oxidation cascades leading to the production of oxylipins such as jasmonic acid, which can induce several secondary metabolites, especially terpenes and sesquiterpenes, as plant defenses [50].

## 5. Conclusions

The *G. candidum* was first injected to into *A. sinensis* to stimulate compound synthesis for agarwood formation. Significant compound formation for horizontal color change was observed between the N group and the W, BR, and B groups. The changes in the main compounds between the N group and the W group demonstrated an early response against *G. candidum* invasion. RP-HPLC analysis demonstrated common compounds in the fresh wood during microbial invasion and in two dry agarwood groups. These compounds may be used as standards for agarwood formation. The ratio of terpenes/phenolics differed inversely between fresh wood and dry agarwood. Furthermore, *A. sinensis* accumulated terpenoids in the BR group and alkaloids in the B group. The vertical compound levels were the highest in the lower part (L6, L5, and L4) near the injection site, but differences in the main compounds were not found among the six layers. In conclusion, the phytocompounds from *A. sinensis* differ from those of previous reports and may have other medicinal functions for animal and human health. 

## Figures and Tables

**Table 1:** Phytocompound changes among different color parts in horizontal layer detected at wavelengths of 230 nm and 280 nm.

Peak No.	Retention Time	N		W		BR		B		Out		In	
		Area	Ratio	Area	Ratio	Area	Ratio	rea	Ratio	Area	Ratio	Area	Ratio
230 nm													
1	20.4	1,716,574	1	3,887,660	2.26	887,171	0.52						
2	20.9	4,822,401	1	2,674,558	0.55	1,118,204	0.23			139,832	0.03	240,109	0.05
3	21.7	4,277,978	1	5,298,952	1.24	3,332,879	0.78	248,852	0.06	86,746	0.02	429,904	0.1
4	27.8									2,318,365	1	4,203,646	1.81
5	29.9	318,670	1	3,092,236	9.7	17,082,604	53.6	2,666,687	8.36	465,954	1.46	1,125,677	3.53
6	30.6	298,343	1	385,297	1.29	542,284	1.82			3,548,927	11.9	2,310,060	7.74
7	31.9			1,559,666	1	2,939,320	1.88			4,841,456	3.1	10,412,672	6.68
8	32.7	311,156	1	4,838,049	15.54	9,104,716	29.26	390,431	1.25	815,019	2.62		
9	33.1	1,639,098	1									1,142,464	0.7
10	34.9			1,186,293	1	10,139,084	8.54	854,797	0.72	7,357,199	6.2	9,698,256	8.18
11	35.5	344,849	1	936,099	2.71	1,437,922	4.17						
12	36.2	139,672	1	1,183,052	8.47	4,712,759	33.74	101,586	0.73	1,019,686	7.3	1,355,914	9.71
13	36.6			165,573	1	339,462	2.05	1,237,104	7.47	441,221	2.66	1,808,474	10.92
14	37.3	56,884	1	150,481	2.65	713,838	12.55			1,698,236	29.85	9,463,337	166.36
15	38.7									2,835,240	1		
16	40.9							249,206	1	98,974	0.4	910,604	3.65
280 nm													
1	10.6	362,536	1	607,812	1.68	214,106	0.59						
2	15.2	92,002	1	814,216	8.85	117,564	1.28			72,532	0.79		
3	15.9	128,253	1	206,993	1.61	72,779	0.57			10,475,534	81.68	2,039,704	15.9
4	20.9	3,001,496	1	287,607	0.1	356,796	0.12			1,657,796	0.55	1,083,141	0.36
5	21.7	1,279,119	1	959,945	0.75	737,287	0.58			505,291	0.4	1,554,411	1.22
6	29.9			434,630	1	3,476,545	8	135,586	0.32	1,832,742	4.22	3,431,303	7.89
7	32.1			388,427	1	985,399	2.54			23,089,768	59.44	43,793,661	112.75
8	32.7	79,164	1	1,646,544	20.8	3,568,234	45.07	68,782	0.87	3,108,963	39.27	3,489,338	44.08
9	34.9			211,275	1	2,149,618	10.17	170,786	0.81	19,210,528	90.92	24,649,599	116.67
10	36.2	8167	1	190,458	23.32	763,980	93.54						
11	37.2					198,330	1			7,361,700	37.12	40,679,299	205.11
12	38.7									16,685,800	1	20,670,580	1.24
13	39.6			108,675	1	217,618	2					1,145,656	10.54
14	40.9							83,378	1				

N: non-injected wood, W: white part, BR: brown part, B: black part, Out: agarwood from market, and In: agarwood from same plantation.

**Table 2:** Compound differences among three color samples from layers L1 to L6.

RT	W						BR						B					
	L1	L2	L3	L4	L5	L6	L1	L2	L3	L4	L5	L6	L1	L2	L3	L4	L5	L6
**230 nm**																		
**14.9**							**0.8**	**0.8**	**0.8**	**1.1**	**1.1**	**1.2**	**2.0**	**1.1**	**1.2**	**1.9**	**2.2**	**2.5**
**20.4**	**3.8**	**3.5**	**3.2**	**3.6**	**3.7**	**4.6**												
**20.9**	**2.6**	**2.9**	**2.2**	**2.4**	**2.4**	**2.5**												
**21.7**	**5.1**	**5.2**	**5.1**	**4.4**	**4.7**	**5.5**							**3.1**	**4.8**	**5.0**	**5.2**	**5.2**	**6.0**
**22.3**													**2.8**	**3.4**	**4.4**	**4.2**	**2.7**	**2.9**
**23.1**													**1.3**	**2.6**	**3.2**	**2.7**	**3.1**	**3.7**
**25.9**													**4.2**	**4.1**	**4.9**	**4.4**	**4.4**	**4.0**
**29.9**	**3.0**	**4.4**	**7.2**	**3.8**	**3.8**	**3.5**	**15.1**	**16.2**	**17.8**	**15.3**	**13.2**	**15.4**	**8.2**	**9.7**	**8.0**	**8.7**	**8.7**	**5.9**
**32.6**	**4.7**	**5.2**	**6.1**	**5.0**	**5.4**	**5.3**	**8.1**	**7.7**	**6.8**	**8.9**	**8.8**	**8.4**	**4.8**	**4.1**	**2.4**	**2.9**	**3.3**	**3.1**
**33.8**	**1.6**	**1.9**	**1.6**	**1.3**	**1.3**	**1.6**												
**34.4**	**1.4**	**1.9**	**2.1**	**1.5**	**1.5**	**1.7**												
**34.9**	**1.2**	**2.1**	**4.6**	**1.8**	**1.8**	**1.3**	**9.0**	**10.5**	**14.0**	**9.3**	**10.0**	**9.7**	**10.5**	**8.8**	**9.0**	**8.8**	**8.0**	**4.6**
**35.4**	**0.9**	**1.2**	**1.0**	**1.0**	**1.0**	**1.0**												
**36.2**	**1.1**	**0.3**	**2.3**	**1.5**	**1.4**	**1.4**	**4.2**	**4.7**	**0.3**	**5.3**	**5.2**	**4.6**						
**36.6**													**15.2**	**4.0**	**3.6**	**2.3**	**3.4**	**4.2**
**40.9**													**3.1**	**2.1**	**1.4**	**2.6**	**2.3**	**2.8**
**280 nm**																		
**3.2**													**4.4**	**1.0**	**1.3**	**1.5**	**1.1**	
**14.7**	**1.5**	**1.2**	**2.7**	**1.2**	**1.5**	**1.2**												
**15.2**	**4.5**	**2.6**	**3.5**	**3.5**	**3.4**	**2.9**												
**15.5**	**2.5**	**1.8**	**2.2**	**2.2**	**2.1**	**2.0**												
**16.2**	**1.7**	**1.6**	**1.5**	**1.9**	**1.7**	**1.1**												
**17.3**	**3.4**	**1.8**	**2.2**	**1.1**	**2.2**	**1.5**												
**17.7**	**1.7**	**1.6**	**1.3**	**2.3**	**1.4**	**2.5**												
**18.5**	**3.1**	**2.1**	**3.0**	**1.3**	**2.6**	**1.6**												
**18.9**	**1.6**	**1.56**	**1.6**	**2.7**	**1.6**	**2.1**												
**19.5**	**2.4**	**2.3**	**2.4**	**1.6**	**2.8**	**2.8**												
**20.4**	**1.5**	**1.3**	**1.2**	**1.6**	**1.6**	**1.6**	**1.5**	**1.3**	**1.2**	**1.6**	**1.6**	**1.6**	**1.5**	**2.9**	**3.6**	**1.6**	**2.5**	**2.1**
**20.9**	**1.6**	**2.6**	**1.7**	**1.7**	**1.6**	**1.7**												
**21.2**	**1.1**	**1.3**	**1.4**	**1.5**	**4.4**	**1.5**								**1.1**	**2.4**	**2.4**	**2.3**	**2.6**
**21.7**	**5.3**	**4.3**	**4.7**	**4.7**	**1.5**	**4.4**							**3.1**	**4.8**	**5.0**	**5.2**	**5.2**	**6**
**22.3**													**2.8**	**23.4**	**4.4**	**4.2**	**2.7**	**2.9**
**23.1**														**1.3**	**2.6**	**3.2**	**2.7**	**3.1**
**23.5**	**1.8**	**2.7**	**2.6**	**28**	**2.6**	**2.8**	**2.3**	**2.0**	**1.8**	**1.8**	**1.8**	**2.0**						
**24.0**	**1.2**	**1.9**	**1.7**	**1.8**	**1.8**	**1.8**	**2.6**	**2.1**	**1.9**	**1.8**	**1.8**	**2.2**						
**24.3**							**1.4**	**1.2**	**1.6**	**1.8**	**1.3**	**1.7**						
**25.0**							**1.4**	**2.8**	**3.2**	**2.7**	**2.7**	**2.4**		**5.2**	**4.9**	**2.2**	**2.4**	**2.1**
**25.7**	**1.7**	**1.9**	**1.8**	**1.7**	**1.8**	**1.9**	**2.7**	**2**	**1.9**	**2.0**	**2.0**	**2.4**	**3.7**	**1.8**	**6.9**	**1.5**	**1.7**	**1.8**
**26.0**	**1.7**	**2.2**	**2.5**	**2.4**	**2.2**	**2.1**	**4.6**	**4.2**	**4.3**	**4.0**	**3.8**	**4.9**		**4.1**	**2.4**	**4.4**	**4.4**	**4.0**
**26.4**	**1.1**	**2.0**	**1.7**	**1.7**	**1.8**	**1.9**	**2.1**	**2.1**	**1.5**	**2.1**	**2**	**2.3**						
**28.2**	**1.6**	**2.3**	**1.8**	**1.7**	**2.0**	**2.1**												
**28.7**	**1.6**	**1.8**	**1.7**	**1.6**	**1.7**	**1.7**												
**29.2**							**2.0**	**1.7**	**1.7**	**1.9**	**1.9**	**1.9**						
**29.4**	**1.4**	**1.5**	**1.6**	**1.1**	**1.3**	**1.4**	**4.8**	**4.7**	**4.6**	**4.2**	**4.2**	**5.1**						
**29.9**	**2.4**	**3.1**	**5.0**	**2.8**	**3.0**	**2.2**	**11.4**	**13.3**	**16.2**	**12.1**	**12.6**	**15.3**	**8.2**	**9.7**	**8.0**	**8.7**	**8.7**	**5.9**
**32.1**	**2.2**	**2.0**	**1.7**	**1.6**	**1.7**	**1.8**	**3.2**	**3.3**	**3.2**	**3.4**	**3.3**	**3.2**						
**32.6**	**9.2**	**6.3**	**8.1**	**8.4**	**7.6**	**6.1**	**11.7**	**11.3**	**11.5**	**13.4**	**13.7**	**15.8**	**4.8**	**4.1**	**1.9**	**1.4**	**3.3**	**3.1**
**33.8**	**1.7**	**1.5**	**1.2**	**1.1**	**1.1**	**1.2**	**2.2**	**1.7**	**1.4**	**1.9**	**1.8**	**1.5**						
**34.4**	**1.4**	**1.2**	**1.5**	**1.2**	**1.08**	**1.06**							**1.4**	**1.8**	**9.0**	**8.8**	**1.4**	**1.1**
**34.9**							**7.0**	**8.9**	**12.9**	**7.5**	**8.9**	**10.2**	**10.5**	**8.8**	**1.3**	**1.8**	**8.0**	**4.6**
**35.5**							**1.1**	**1.3**	**1.3**	**1.5**	**1.5**	**1.4**						
**36.2**							**2.5**	**3.0**	**3.6**	**3.4**	**3.2**	**3.6**	**1.3**	**2.3**		**2.3**	**1.9**	**1.2**
**36.6**													**15.2**	**4.0**	**3.6**		**3.4**	**4.2**
**40.9**													**3.1**	**2.1**	**1.2**	**2.6**	**2.3**	**2.8**

**Table 3:** Total peak area and comparison of horizontal and vertical parts under 230 nm and 280 nm.

Wavelength	N	W	BR	B	Out	In
230 nm	72,846,065	121,603,884	138,175,507	32,277,741	40,448,346	66,619,765
	80.33%/1	77.86%/1.7	80.0%/1.9	88.77%/0.44	15.59%/0.56	31.14%/0.91
280 nm	17,826,203	34,570,769	34,542,961	4,083,881	218,965,220	147,339,123
	19.65%/1	22.14%/1.9	20.0%/1.9	11.23%/0.23	84.41%/12.3	68.86%/8.27
Total	90,672,268	156,174,653	172,718,468	36,361,622	259,413,566	213,958,888
	9.76%/1	16.81%/1.59	18.59%/1.9	3.91%/0.4	27.91%/2.86	23.02%/2.36
Wavelength	L6-BR	L5-BR	L4-BR	L3-BR	L2-BR	L1-BR
230 nm	93,637,794	143,705,044	94,324,220	52,084,536	124,390,618	74,809,914
	78.09%/1	80.31%/1.53	81.19%/1.0	81.69%/0.56	78.02%/1.33	81.93%/0.78
280 nm	26,267,900	35,241,665	21,856,278	11,672,573	35,034,461	16,497,150
	21.91%/1	19.69%/1.34	18.81%/0.83	18.31%/0.44	21.98%/1.33	18.07%/0.63
Total	119,905,694	17,8946,709	116,180,498	63,757,109	159,425,079	91,307,064
	16.4%/1	24.5%/1.49	15.9%/0.97	8.7%/0.53	21.85%/1.33	12.52%/0.76

**Table 4:** The top three compounds in compound groups in horizontal color parts.

Category	Retention Time	Formulas	Horizontal Trunk (%)				Upper Branch BR Part (%)					
			N	White	BR	B	L6	L5	L4	L3	L2	L1
Sequiterpenes	12.27	C_15_H_26_O		0.01	0.31		0.72	0.70	0.32	0.25	0.39	0.25
	13.86	C_15_H_28_O_2_			(0.12)^1^	0.05	(0.30)	(0.22)	(0.10)	(0.11)	(0.13)	0.12
	15.74	C_15_H_26_O			0.23		0.56	0.48	0.22	0.25	0.33	0.15
	16.94	C_15_H_24_			0.17	0.01						
	17.11	C_15_H_24_					0.73	0.68				
	17.57	C_15_H_29_O_2_							(0.11)	0.12	0.11	
	18.13	C_15_H_22_O_2_						(0.35)	0.30			
Chromones	10.9	C_10_H_8_O_2_						0.13	0.09	0.06	0.07	0.06
	20.74	C_11_H_10_O_3_	0.22									
	22.82	C_17_H_24_O_4_			0.04	0.74	0.32				0.05	
	23.48	C_16_H_14_O_4_	0.11			(0.02)	1.27	1.98	1.51	1.53		
	24.01	C_18_H_16_O_3_			0.74	(0.08)						
	24.38	C_18_H_18_O_3_		0.48	1.26	0.5	1.00	1.29	3.40	0.42	1.90	2.68
	26.34	C_17_H_14_O_5_	0.62	1.41		0.16						
	27.02	C_16_H_12_O_5_		0.42								
Terpenoids	8	C_10_H_12_O		0.04	0.41		1.13	0.83	0.37	0.40	0.49	0.29
	8.27	C_10_H_16_O_2_				0.48						
	8.33	C_10_H_12_O_2_					(0.16)	(0.16)	(0.07)	0.06	0.09	
	8.77	C_9_H_10_O_2_		0.05								
	10.55	C_10_H_18_O_3_				0.51						
	13.12	C_10_H_12_O_3_	0.43	0.90	0.26	0.13	0.8	0.52	0.26	0.15	0.25	0.27
	14.3	C_20_H_28_O_2_					(0.23)	0.24				
	16.98	C_20_H_28_O_6_			0.68		0.96					
Alkaloids	9.33	C_6_H_9_NO_3_	0.08									
	11.16	C_13_H_21_NO_2_						0.10	0.05			
	17.28	C_8_H_16_N_2_O_7_			1.28		1.79	1.83				
	23.58	C_18_H_27_NO_3_	2.29									
	23.97	C_14_H_14_C1_2_N_2_			1.19	0.54	0.74				1.73	2.59
	24.73	C_20_H_25_NO	0.04	1.16	1	4.57				0.38	0.42	3.45
	25.66	C_23_H_23_NO		1.16								
	25.83	C_17_H_14_N_2_O_4_		2.20		1.07						
	35.68	C_23_H_23_NO										1.23
Aromatic compounds	4.59	C_7_H_6_O		(0.04)	0.27	(0.06)	0.70	0.51	0.28	0.30	0.31	0.22
	8.91	C_9_H_10_O_2_					(0.48)	(0.41)			0.11	
	9.13	C_8_H_10_O_3_	0.06	(0.09)								
	10.56	C_11_H_14_O_2_					(0.21)	(0.15)	(0.06)	(0.05)	0.08	0.05
	10.63	C_14_H_22_O	0.09	(0.09)	(0.08)	(0.11)						
	11.5	C_9_H_12_O_4_	0.13	(0.15)								
	19.15	C_18_H_27_NO_3_		2.28								
	19.29	C_17_H_16_O						0.79	0.23	0.31		0.17
	20.42	C_15_H_13_IO_2_			(0.20)	0.20			0.28			
	20.43	C_17_H_18_O			0.39	0.23	0.69	0.63		0.34		
	21.54	C_17_H_14_O_2_		(0.68)	0.71	0.79	1.01					
	24.72	C_20_H_25_NO		0.85								
	25.67	C_8_H_10_S_2_		1.13								
Fatty acids	16.02	C_17_H_34_O_2_	0.13	(0.12)	(0.25)	(0.18)		0.55	0.34	0.19	0.21	0.14
	16.58	C_16_H_32_O_2_	0.13	0.72	1.03	0.83	1.58	1.33				
	17.93	C_22_H_34_O_2_					(0.51)	(0.20)	(0.29)	0.19		0.23
	18.33	C_19_H_36_O_2_	0.13	(0.25)	0.43	(0.35)	1.20	1.02	0.50	0.31	0.34	0.29
	18.75	C_18_H_34_O_2_	(0.02)	1.75	(0.29)	0.79						
	24.46	C_18_H_33_ClO				0.53						
	24.47	C_21_H_40_O_4_		(0.19)	0.41							
	24.52	C_21_H_34_O_2_		0.59					0.58		0.58	
Others	10.06	C_4_H_9_NO_5_	1.19	(0.42)								
	11.67	C_6_H_12_O_4_	0.25									
	11.85	C_8_H_16_O_6_		4.57		(0.08)						
	16.58	C_12_H_22_O_11_		9.46	0.74					0.86	0.92	1.38
	16.63	C_6_H_12_O_6_			1.24				1.76			

## Data Availability

The datasets used in the current study are available from the corresponding author on reasonable request.

## Supplementary Materials

The following supporting information can be downloaded at: https://www.mdpi.com/article/10.3390/life13112147/s1, Table S1. GC/MS analysis of compound changes among horizontal color parts. Table S2. GC/MS analysis of vertical compound change in brown part of agarwood.

## References

[1] Mo Q., Fan C., Zhou G., Fu H., Wang Y. (2019). Composition variation of agar-wood-associated microbial communities from *Aquilaria sinensis*. bioRxiv.

[2] Gunn B., Stevens P., Singadan M., Sunari L., Chatterton P. (2004). Eaglewood in Papua New Guinea.

[3] Kalra R., Kaushik N. (2017). A review of chemistry, quality and analysis of infected agar-wood tree (*Aquilaria* sp.). Phytochem. Rev..

[4] Lee S.Y., Turjaman M., Mohamed R. (2018). Phylogenetic relatedness of several agar-wood-producing taxa (*Thymelaeaceae*) from Indonesia. Trop. Life Sci. Res..

[5] Dwianto W., Kusumah S., Darmawan T., Amin Y., Bahanawan A., Pramasari D., Lestari E., Himmi S., Hermiati E., Fatriasari W. (2019). Anatomical observation and characteri-zation on basic properties of agarwood (Gaharu) as an Appendix II CITES. IOP Conference Series: Earth and Environmental Science.

[6] Tan C.S., Isa N.M., Ismail I., Zainal Z. (2019). Agarwood induction: Current developments and future perspectives. Front. Plant Sci..

[7] Naziz P.S., Das R., Sen S. (2019). The scent of stress: Evidence from the unique fragrance of agarwood. Front. Plant Sci..

[8] Ahmed D.T., Mohammed M., Masaad A.M., Tajuddin S.N. (2017). Investigation of agarwood compounds in *Aquilaria malaccensis* & *Aquilaria rostrata* chipwood by using solid phase microextraction. Biomed. J. Sci. Tech. Res..

[9] Novriyanti E., Santosa E., Syafii W., Turjaman M., Sitepu I.R. (2010). Anti fungal activity of wood extract of *Aquilaria crassna* Pierre ex Lecomte against agarwood-inducing fungi, *Fusarium solani*. Indon. J. For. Res..

[10] Modzelewska A., Sur S., Kumar S.K., Khan S.R. (2005). Sesquiterpenes: Natural products that decrease cancer growth. Curr. Med. Chem. Anticancer Agents.

[11] Amirkia V., Heinrich M. (2014). Alkaloids as drug leads–A predictive structural and biodiversity-based analysis. Phytochem. Lett..

[12] Khan H. (2016). Berberine: As a therapeutic target for treating obese diabetes. J. Diabetes Res. Therap..

[13] Chhipa H., Kaushik N. (2017). Fungal and bacterial diversity isolated from *Aquilaria malaccensis* tree and soil, induces agarospirol formation within 3 months after artificial in-fection. Front. Microbiol..

[14] Ocampo L.Q., Stahr M. (2018). Geotrichum Sour Rot of Sweetpotato Vegetable Pathology Factsheets.

[15] Liu J., Zhang X., Yang J., Zhou J., Yuan Y., Jiang C., Chi X., Huang L. (2019). Agarwood wound locations provide insight into the association between fungal diversity and volatile compounds in *Aquilaria sinensis*. R. Soc. Open Sci..

[16] Wang M.-R., Li W., Luo S., Zhao X., Ma C.-H., Liu S.-X. (2018). GC-MS Study of the chemical components of different *Aquilaria sinensis* (Lour.) Gilgorgans and Agarwood from different Asian countries. Molecules.

[17] Siriangkhawut W., Kaewboo I. (2013). Ultrasonic extraction method for alizarin from roots of *Morinda citrifolia*. Anal. Chem. Indian J..

[18] Hendra H., Moeljopawiro S., Nuringtyas T.R. (2016). Antioxidant and antibacterial activi-ties of agarwood (*Aquilaria malaccensis* Lamk.) leaves. AIP Conference Proceedings.

[19] Negi J.S., Singh P., Pant G.J.N., Rawat M. (2011). High-performance liquid chromatography analysis of plant saponins: An update 2005–2010. Pharmacogn. Rev..

[20] Cole M.D. (2003). The Analysis of Controlled Substances.

[21] Zhang A., Wan L., Wu C., Fang Y., Han G., Li H., Zhang Z., Wang H. (2013). Simultaneous determination of 14 phenolic compounds in grape canes by HPLC-DAD-UV using wavelength switching detection. Molecules.

[22] Weaver L.M., Herrmann K.M. (1997). Dynamics of the shikimate pathway in plants. Trends Plant Sci..

[23] Hashim Y.Z.H.-Y., Ismail N.I., Abbas P. (2014). Analysis of chemical compounds of agar-wood oil from different species by gas chromatography mass spectrometry (GCMS). IIUM Eng. J..

[24] Jones F.R. (1928). Development of the bacteria causing wilt in the Alfalfa plant as influenced by growth and winter injury. J. Agric. Res..

[25] Blanchette R. (1992). Anatomical responses of xylem to injury and invasion by fungi. Defense Mechanisms of Woody Plants against Fungi.

[26] Zhang Z., Han X.M., Wei J.H., Xue J., Yang Y., Liang L., Li X.J., Guo Q.M., Xu Y.H., Gao Z.H. (2014). Compositions and antifungal activities of essential oils from agarwood of *Aquilaria sinensis* (Lour.) Gilg induced by *Lasiodiplodia theobromae* (Pat.) Griffon. & Maubl. J. Braz. Chem. Soc..

[27] Lin D., Xiao M., Zhao J., Li Z., Xing B., Li X., Kong M., Li L., Zhang Q., Liu Y. (2016). An overview of plant phenolic compounds and their importance in human nutrition and management of type 2 diabetes. Molecules.

[28] Mandal S.M., Chakraborty D., Dey S. (2010). Phenolic acids act as signaling molecules in plant-microbe symbioses. Plant Signal. Behav..

[29] Novriyanti E., Santosa E. (2011). The role of phenolics in agarwood formation of *Aquilaria crassna* Pierre ex Lecomte and *Aquilaria microcarpa* Baill Trees. Indon. J. For. Res..

[30] López-Sampson A., Page T. (2018). History of use and trade of agarwood. Econ. Bot..

[31] Ismail N., Ali N.A.M., Jamil M., Rahiman M.H.F., Tajuddin S.N., Taib M.N. (2014). A review study of agarwood oil and its quality analysis. J. Teknol..

[32] Liu Y., Chen H., Yang Y., Zhang Z., Wei J., Meng H., Chen W., Feng J., Gan B., Chen X. (2013). Whole-tree agarwood-inducing technique: An efficient novel technique for producing high-quality agarwood in cultivated *Aquilaria sinensis* trees. Molecules.

[33] Mohamed R., Lee S.Y. (2016). Keeping up appearances: Agarwood grades and quality. Agarwood.

[34] Singh B., Sharma R.A. (2015). Plant terpenes: Defense responses, phylogenetic analysis, regulation and clinical applications. 3 Biotech.

[35] Faizal A., Esyanti R.R., Aulianisa E.N., Santoso E., Turjaman M. (2017). Formation of agarwood from *Aquilaria malaccensis* in response to inoculation of local strains of *Fusarium solani*. Trees.

[36] Sinha S., Sandhu K., Bisht N., Naliwal T., Saini I., Kaushik P. (2019). Ascertaining the paradigm of secondary metabolism enhancement through gene level modification in therapeutic plants. J. Young Pharm..

[37] Setyorini S., Yusnawan E. (2016). The increase of secondary metabolite in legumes as a response of biotic stress. Iptek Tanam. Pangan.

[38] Ye W., He X., Wu H., Wang L., Zhang W., Fan Y., Li H., Liu T., Gao X. (2018). Identification and characterization of a novel sesquiterpene synthase from *Aquilaria sinensis*: An important gene for agarwood formation. Int. J. Biol. Macromol..

[39] Nasution R., Muhabbah N., Helwati H., Bahi M., Marianne M., Amna U. (2020). Isolation of lupeol acetate from fruit peels of *Artocarpus camansi*. J. Adv. Pharm. Technol. Res..

[40] Yang J., Duan G., Li C., Liu L., Han G., Zhang Y., Wang C. (2019). The crosstalks between jasmonic acid and other plant hormone signaling highlight the involvement of jasmonic acid as a core component in plant response to biotic and abiotic stresses. Front. Plant Sci..

[41] Xu Y., Zhang Z., Wang M., Wei J., Chen H., Gao Z., Sui C., Luo H., Zhang X., Yang Y. (2013). Identification of genes related to agarwood formation: Transcriptome analysis of healthy and wounded tissues of *Aquilaria sinensis*. BMC Genom..

[42] Chen X., Zhu X., Feng M., Zhong Z., Zhou X., Chen X., Ye W., Zhang W., Gao X. (2017). Relationship between expression of chalcone synthase genes and chromones in artificial agarwood induced by formic acid stimulation combined with *Fusarium* sp. A2 inoculation. Molecules.

[43] Aung K., Jiang Y., He S.Y. (2018). The role of water in plant–microbe interactions. Plant J..

[44] Rasool S., Mohamed R. (2016). Understanding agarwood formation and its challenges. Agarwood.

[45] Yan T., Yang S., Chen Y., Wang Q., Li G. (2019). Chemical profiles of cultivated Agarwood induced by different techniques. Molecules.

[46] Yin Y., Jiao L., Dong M., Jiang X., Zhang S. (2016). Wood resources, identification, and utilization of agarwood in China. Agarwood.

[47] Naef R. (2011). The volatile and semi-volatile constituents of agarwood, the infected heart-wood of *Aquilaria* species: A review. Flavour Fragr. J..

[48] Hishamuddin M.S., Lee S.Y., Isa N.M., Lamasudin D.U., Abidin S.A.Z., Mohamed R. (2019). Time-based LC-MS/MS analysis provides insights into early responses to mechanical wounding, a major trigger to agarwood formation in *Aquilaria malaccensis* Lam. RSC Adv..

[49] Adibah K.Z.M., Azzreena M.A. (2019). Plant toxins: Alkaloids and their toxicities. GSC Biol. Pharmaceut. Sci..

[50] Sen S., Dehingia M., Talukdar N.C., Khan M. (2017). Chemometric analysis reveals links in the formation of fragrant bio-molecules during agarwood (*Aquilaria malaccensis*) and fungal interactions. Sci. Rep..

