# Supplementary material for: Progressive Vertical and Horizontal Phytocompound Changes during Agarwood Formation in Aquilaria sinensis after Geotrichum candidum Injection
Source: Life (Basel). 2023 Oct 31;13(11):2147. doi: 10.3390/life13112147 (PMC10672404; doi:10.3390/life13112147)
Supplement: Supplementary file 1 [file life-13-02147-s001.zip › life-2549694-supplementary.pdf]

Supplkementary files

Table S1. GC/MS analysis of compound changes among horizontal color parts

Sesquiterpene							
**Structure**	**Formula**	**Compound name**	**RT**	**N**	**W**	**BR**	**B**
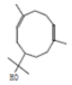	C15H26O	3,7-Cyclodecadiene-1-methanol, .alpha.,.alpha.,4,8-tetramethyl-, [s-(Z,Z)] $$ Hedycaryol	11.092	0.00	0.00	0.01	0.00
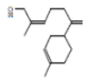	C15H24O	Lanceol, cis	11.425	0.00	0.00	0.05	0.00
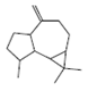	C15H24	1H-Cycloprop[e]azulene, decahydro-1,1,7-trimethyl-4-methylene-, [1aR-(1a.alpha.,4a.alpha.,7.alpha.,7a.beta.,7b.alpha.)	12.092	0.00	0.00	0.10	0.00
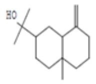	C15H26O	2-Naphthalenemethanol, decahydro-.alpha.,.alpha.,4a-trimethyl-8-methylene-, [2R-(2.alpha.,4a.alpha.,8a.beta.)]-	12.267	0.00	0.01	0.31	0.00
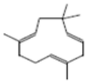	C15H24	Humulene	12.875	0.00	0.00	0.12	0.00
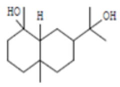	C15H28O2	(1R,4aR,7R,8aR)-7-(2-Hydroxypropan-2-yl)-1,4a-dimethyldecahydronaphthalen-1-ol	13.858	0.00	0.00	0.12	0.05
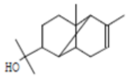	C15H24O	Tricyclo[4.4.0.0(2,7)]dec-8-ene-3-methanol, .alpha.,.alpha.,6,8-tetramethyl-, stereoisomer	15.208	0.00	0.00	0.13	0.00
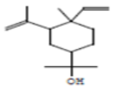	C15H26O	Cyclohexanemethanol, 4-ethenyl-.alpha.,.alpha.,4-trimethyl-3-(1-methylethenyl)-, [1R-(1.alpha.,3.alpha.,4.beta.)]-	15.742	0.00	0.00	0.23	0.02
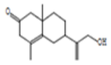	C15H26O	6-(1-Hydroxymethylvinyl)-4,8a-dimethyl-3,5,6,7,8,8a-hexahydro-1H-naphthalen-2-one	16.433	0.00	0.00	0.08	0.00
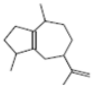	C15H24	Azulene, 1,2,3,4,5,6,7,8-octahydro-1,4-dimethyl-7-(1-methylethenyl)-, [1S-(1.alpha.,4.alpha.,7.alpha.)]-	16.942	0.00	0.00	0.17	0.01
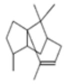	C15H24	epi-.alpha.-Patchoulene	17.108	0.00	0.00	0.16	0.00
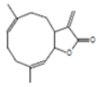	C15H20O2	Germacra-1(10),4,11(13)-trien-12-oic acid, 6.alpha.-hydroxy-, .gamma.-lactone, (E,E)-	17.567	0.00	0.00	0.04	0.00
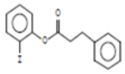	C15H13IO2	2-Iodophenyl-.beta.-phenylpropionate	20.425	0.00	0.00	0.05	0.00
		TOTAL		0.00	0.01	1.57	0.07
				1.65			

Chromone

Structure	Formula	Compound name	RT	N	W	BW	B
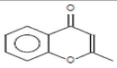	C10H8O2	4+AJ21:AJ44H-1-Benzopyran-4-one, 2-methyl-	10.9	0.00	0.00	0.05	0.00
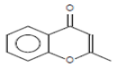	C10H8O2	4H-1-Benzopyran-4-one, 2-methyl-	10.9	0.00	0.00	0.00	0.02
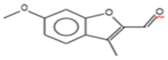	C11H10O3	6-Methoxy-3-methyl-2-benzofurancarbaldehyde	20.742	0.22	0.00	0.00	0.03
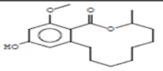	C17H24O4	2-Hydroxy-4-methoxy-7-methyl-7,8,9,10,11,12,13,14-octahydro-6-oxabenzocyclododecen-5-one	22.817	0.00	0.00	0.04	0.74
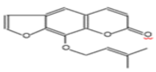	C16H14O4	7H-Furo[3,2-g][1]benzopyran-7-one, 9-[(3-methyl-2-butenyl)oxy]-	23.483	0.11	0.00	0.00	0.02
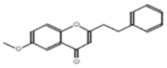	C18H16O3	6-Methoxy-2-phenethyl-4H-chromen-4-one	24.008	0.00	0.00	0.74	0.08
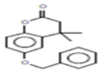	C18H18O3	Coumarin, 6-benzyloxy-3,4-dihydro-4,4-dimethyl-	24.375	0.00	0.48	1.26	0.50
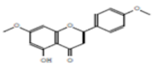	C17H14O5	4H-1-Benzopyran-4-one, 5-hydroxy-7-methoxy-2-(4-methoxyphenyl)-	26.342	0.62	1.41	0.00	0.16
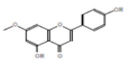	C16H12O5	Genkwanin	27.017	0.00	0.42	0.00	0.00
		TOTAL		0.96	2.31	2.09	1.55
						6.91	

Terpenoid

Structure	Formula	Compounds	RT	N	W	BW	B
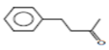	C10H12O	2-Butanone, 4-phenyl-	8	0.00	0.04	0.41	0.00
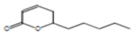	C10H16O2	2H-Pyran-2-one, 5,6-dihydro-6-pentyl	8.267	0.00	0.00	0.00	0.48
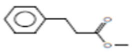	C10H12O2	Benzenepropanoic acid, methyl ester	8.333	0.00	0.00	0.07	0.00
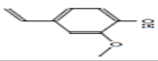	C9H10O2	2-Methoxy-4-vinylphenol	8.767	0.00	0.05	0.00	0.00
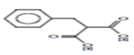	C10H10O4	Benzylmalonic acid	8.9	0.00	0.00	0.06	0.06
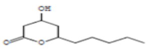	C10H18O3	2H-Pyran-2-one, tetrahydro-4-hydroxy-6-pentyl-	10.55	0.00	0.00	0.00	0.51
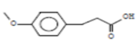	C10H12O3	3-(4-Methoxyphenyl)propionic acid	11.258	0.00	0.00	0.12	0.00
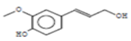	C10H12O3	4-((1E)-3-Hydroxy-1-propenyl)-2-methoxyphenol	13.1	0.43	0.90	0.26	0.13
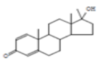	C20H28O2	Androsta-1,4-dien-3-one, 17-hydroxy-17-methyl-, (17.alpha.)-	14.3	0.00	0.00	0.16	0.00
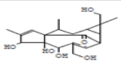	C20H28O6	1H-2,8a-Methanocyclopenta[a]cyclopropa[e]cyclodecen-11-one, 1a,2,5,5a,6,9,10,10a-octahydro-5,5a,6-trihydroxy-1,4-	16.95	0.00	0.00	0.68	0.06
		TOTAL		0.43	0.99	1.76	1.24
						4.42	

Alkaloid							
**Structure**	**Formula**	**Compounds**	**RT**	**N**	**W**	**BW**	**B**
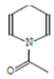	C7H9NO	4(H)-Pyridine, N-acetyl-	5.292	0.00	0.02	0.00	0.00
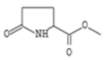	C6H9NO3	DL-Proline, 5-oxo-, methyl ester	9.333	0.08	0.00	0.00	0.00
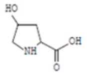	C5H9NO3	Pyrrolidin-4-ol-2-carboxylic acid	9.458	0.00	0.00	0.00	0.43
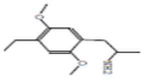	C13H21NO2	2,5-Dimethoxy-4-ethylamphetamine	11.158	0.03	0.13	0.06	0.00
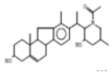	C29H41NO3	N-Acetyl-veratramine	11.775	0.00	0.41	0.00	0.00
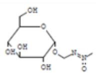	C8H16N2O7	beta.-D-glucosyloxyazoxymethane	17.283	0.00	0.00	1.28	0.00
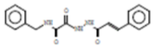	C18H17N3O3	Oxalic acid, monoamide monohydrazide, N-benzyl-N''-(1-oxo-3-phenylprop-2-enyl)-	17.667	0.00	0.00	0.31	0.00
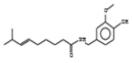	C18H27NO3	Capsaicin	23.575	4.1006	0.00	0.00	0.00
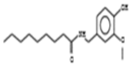	C17H27NO3	Nonivamide	23.75	0.38	0.00	0.00	0.00
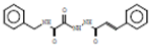	C14H14Cl2N2	2,6-Lutidine-4-[benzylamino]-3,5-dichloro	23.967	0.00	0.65	1.19	0.54
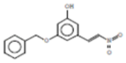	C15H13NO4	benzene-1,2-diol, 4-[2-nitroethenyl]-, 1-O-benzyl(ether)	24.225	0.00	0.00	0.45	0.00
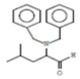	C20H25NO	Pentanal, 2-[bis(phenylmethyl)amino]-4-methyl-	24.725	0.04	1.16	1.00	4.57
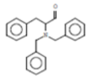	C23H23NO	Benzenepropanal, .alpha.-[bis(phenylmethyl)amino]-	25.658	0.00	1.16	0.87	0.40
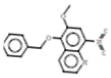	C17H14N2O4	5-Benzyloxy-6-methoxy08nitroquinoline	25.825	0.00	2.20	0.00	1.07
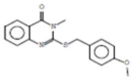	C17H16N2O2S	Quinazolin-4(3H)-one, 2-(4-methoxybenzylthio)-3-methyl	26.075	0.00	0.61	0.00	0.13
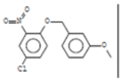	C14H12ClNO4	Benzene, 1-chloro-4-(4-methoxybenzyloxy)-3-nitro-	26.533	0.00	0.92	0.00	0.00
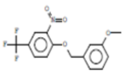	C15H12F3NO4	Benzene, 1-trifluoromethyl-4-(3-methoxybenzyloxy)-3-nitro-	27.733	0.00	0.76	0.00	0.00
TOTAL				4.63	8.02	5.15	7.13
						24.93	

Aromatic compound							
**Structure**	**Formula**	**Compound**	**RT**	**N**	**W**	**BW**	**B**
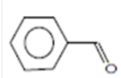	C7H6O	Benzaldehyde	4.592	0.00	0.04	0.27	0.06
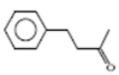	C10H12O	2-Butanone, 4-phenyl-	8.008	0.00	0.05	0.00	0.00
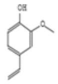	C9H10O2	2-Methoxy-4-vinylphenol	8.767	0.05	0.10	0.00	0.00
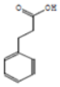	C9H10O2	Hydrocinnamic acid	8.908	0.00	0.00	0.24	0.00
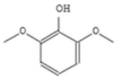	C8H10O3	Phenol, 2,6-dimethoxy-	9.125	0.06	0.09	0.00	0.00
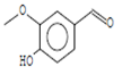	C8H8O3	Vanillin	9.633	0.00	0.06	0.00	0.00
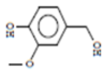	C8H10O3	4-Hydroxy-3-methoxybenzyl alcohol	10.117	0.00	0.05	0.00	0.00
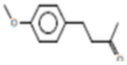	C11H14O2	2-Butanone, 4-(4-methoxyphenyl)-	10.558	0.00	0.00	0.07	0.00
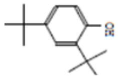	C14H22O	2,4-Di-tert-butylphenol	10.625	0.00	0.00	0.05	0.00
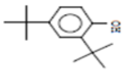	C14H22O	Phenol, 2,4-bis(1,1-dimethylethyl)-	10.625	0.09	0.09	0.08	0.11
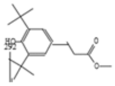	C18H28O3	Benzenepropanoic acid, 3,5-bis(1,1-dimethylethyl)-4-hydroxy-, methyl ester	10.808	0.00	0.00	0.03	0.00
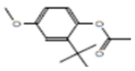	C13H18O3	3-tert-Butyl-4-hydroxyanisole, acetate	11.158	0.00	0.08	0.05	0.00
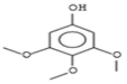	C9H12O4	Phenol, 3,4,5-trimethoxy-	11.5	0.13	0.15	0.00	0.00
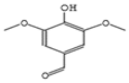	C9H10O4	Benzaldehyde, 4-hydroxy-3,5-dimethoxy-	12.167	0.04	0.09	0.00	0.00
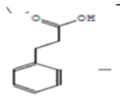	C9H10O2	Hydrocinnamic acid	17.642	0.00	0.00	0.26	0.00

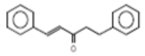	C17H16O	1-Penten-3-one, 1,5-diphenyl-	19.292	0.00	0.07	0.26	0.00
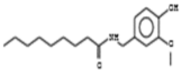	C17H27NO3	Nonivamide	19.642	0.00	0.27	0.00	0.00
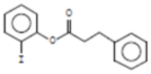	C15H13IO2	2-Iodophenyl-.beta.-phenylpropionate	20.417	0.00	0.00	0.20	0.20
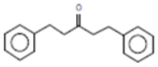	C17H18O	3-Pentanone, 1,5-diphenyl-	20.433	0.00	0.00	0.39	0.23
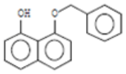	C17H14O2	8-Naphthol, 1-(benzyloxy)-	21.542	0.00	0.68	0.71	0.79
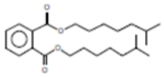	C24H38O4	1,2-Benzenedicarboxylic acid, diisooctyl ester	23.425	0.05	0.00	0.12	0.06
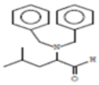	C20H25NO	Pentanal, 2-[bis(phenylmethyl)amino]-4-methyl-	24.717	0.00	0.85	0.00	0.00
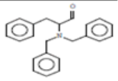	C23H23NO	Benzenepropanal, .alpha.-[bis(phenylmethyl)amino]-	25.658	0.00	0.48	0.00	0.00
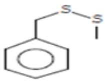	C8H10S2	Benzyl methyl disulfide	25.667	0.00	1.13	0.00	0.00
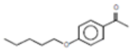	C13H18O2	p-(Pentyloxy)acetophenone	26.9	0.00	0.45	0.00	0.00
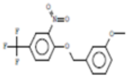	C15H12F3NO4	Benzene, 1-trifluoromethyl-4-(3-methoxybenzyloxy)-3-nitro-	27.725	0.00	0.51	0.00	0.00
TOTAL				0.41	5.25	2.72	1.45
				10.33			

Fatty acid							
**Structure**	**Formula**	**Compound**	**RT**	**N**	**W**	**BW**	**B**
	C19H36O2	11-Octadecenoic acid, methyl ester	18.333	0.13	0.25	0.43	0.35
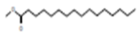	C17H34O2	Hexadecanoic acid, methyl ester	16.017	0.13	0.12	0.25	0.18
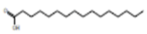	C16H32O2	n-Hexadecanoic acid	16.575	0.13	0.72	1.03	0.83
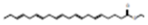	C22H34O2	Ethyl 5,8,11,14,17-icosapentaenoate	17.925	0.00	0.00	0.21	0.00
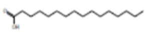	C16H32O2	1-Heptadecanol	18.133	0.00	0.00	0.00	0.05
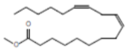	C19H34O2	9,12-Octadecadienoic acid (Z,Z)-, methyl ester	18.283	0.00	0.00	0.31	0.14
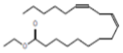	C20H36O2	Linoleic acid ethyl ester	18.283	0.06	0.11	0.00	0.00
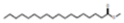	C19H38O2	Octadecanoic acid, methyl ester	18.6	0.00	0.00	0.05	0.08
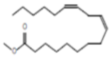	C19H34O2	9,12-Octadecadienoic acid (Z,Z)-	18.692	0.00	0.00	0.00	0.23
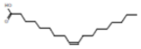	C18H34O2	Oleic Acid	18.75	0.02	1.75	0.29	0.79
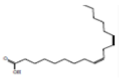	C19H38O2	Octadecanoic acid	18.983	0.00	0.30	0.00	0.15
	C15H30O	Pentadecanal-	22.292	0.00	0.00	0.00	0.04
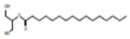	C19H38O4	Hexadecanoic acid, 2-hydroxy-1-(hydroxymethyl)ethyl ester	23.067	0.07	0.13	0.06	0.12
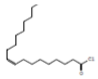	C18H33ClO	Oleoyl chloride	24.458	0.00	0.00	0.00	0.53
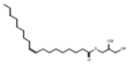	C21H40O4	9-Octadecenoic acid (Z)-, 2,3-dihydroxypropyl ester	24.467	0.00	0.19	0.41	0.00
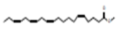	C21H34O2	Methyl (Z)-5,11,14,17-eicosatetraenoate	24.517	0.00	0.59	0.00	0.00
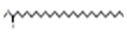	C27H54O2	Hexacosanoic acid	25.925	0.00	0.00	0.00	0.16
TOTAL				0.55	4.16	3.04	3.65
						11.40	

Other							
**Structure**	**Formula**	**Compound name**	**RT**	**N**	**W**	**BW**	**B**
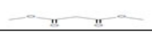	C5H8O4	Propanedioic acid, dimethyl ester	4.067	0.00	0.06	0.00	0.00
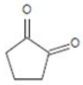	C5H6O2	1,2-Cyclopentanedione	4.092	0.00	0.04	0.00	0.00
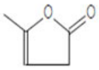	C5H6O2	2(3H)-Furanone, 5-methyl-	4.1	0.00	0.05	0.00	0.00
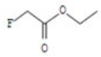	C4H7FO2	Acetic acid, fluoro-, ethyl ester	4.6	0.00	0.00	0.00	0.11
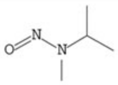	C4H10N2O	2-Propanamine, N-methyl-N-nitroso-	6.708	0.03	0.00	0.00	0.00
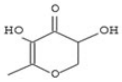	C6H8O4	4H-Pyran-4-one, 2,3-dihydro-3,5-dihydroxy-6-methyl-	6.858	0.05	0.00	0.00	0.00
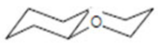	C9H16O	trans-2-Oxabicyclo[4.4.0]decane	7.625	0.00	0.00	0.00	0.06
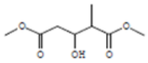	C8H14O5	3-Hydroxy-2-methylglutaric acid dimethyl ester	7.708	0.00	0.08	0.26	0.00
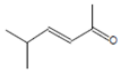	C7H12O	3-Hexen-2-one, 5-methyl-	8.008	0.00	0.00	0.00	0.15
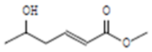	C9H10O2	Hydrocinnamic acid	8.9	0.00	0.00	0.00	0.04
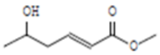	C7H12O3	5-Hydroxy-hex-2-enoic acid, methyl ester	9.075	0.00	0.00	0.00	0.04
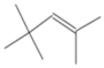	C8H16	2-Pentene, 2,4,4-trimethyl-	9.2	0.15	0.00	0.00	0.00
	C13H28	Tridecane	9.533	0.00	0.00	0.00	0.04
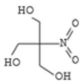	C4H9NO5	1,3-Propanediol, 2-(hydroxymethyl)-2-nitro-	10.058	1.19	0.42	0.00	0.00
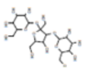	C18H32O16	alpha.-D-Glucopyranoside, O-.alpha.-D-glucopyranosyl-(1.fwdarw.3)-.beta.-D-fructofuranosyl	10.375	0.00	0.00	0.00	0.08

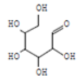	$C_6H_{12}O_6$	D-Allose	10.392	0.00	0.22	0.07	0.00
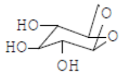	$C_6H_{10}O_5$	1,6-Anhydro-.beta.-D-glucopyranose (levoglucosan)	10.417	0.00	0.29	0.00	0.00
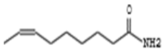	$C_9H_{17}NO$	7-Nonenamide	10.517	0.02	0.00	0.00	0.00
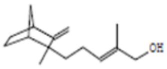	$C_{15}H_{24}O$	Santalol	11.417	0.00	0.00	0.04	0.00
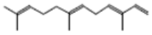	$C_{15}H_{24}$	alpha.-Farnesene	11.433	0.00	0.00	0.20	0.00
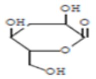	$C_6H_{10}O_5$	3-Deoxy-d-mannoic lactone	11.642	0.00	0.26	0.00	0.00
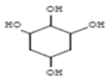	$C_6H_{12}O_4$	1,2,3,5-Cyclohexanetetrol, (1.alpha.,2.beta.,3.alpha.,5.beta.)-	11.667	0.25	0.28	0.00	0.00
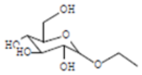	$C_8H_{16}O_6$	Ethyl .alpha.-d-glucopyranoside	11.85	0.00	4.57	0.00	0.08
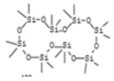	$C_{16}H_{48}O_8Si_8$	Cyclooctasiloxane, hexadecamethyl	12.075	0.21	0.00	0.00	0.00
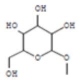	$C_7H_{14}O_6$	beta.-D-Glucopyranoside, methyl	13.192	0.00	0.21	0.00	0.00
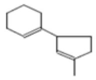	$C_{12}H_{18}$	1-(3-Methyl-cyclopent-2-enyl)-cyclohexene	15.217	0.00	0.00	0.09	0.00
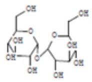	$C_{12}H_{22}O_{11}$	alpha.-D-Glucopyranoside, .alpha.-D-glucopyranosyl	16.575	0.00	9.46	0.74	0.00
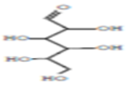	$C_6H_{12}O_6$	D-Galactose	16.625	0.00	0.00	1.24	0.00
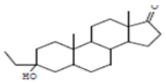	$C_{21}H_{34}O_2$	Androstan-17-one, 3-ethyl-3-hydroxy-, (5.alpha.)-	16.967	0.00	0.00	0.47	0.15
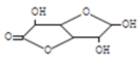	$C_6H_8O_6$	D-Glucurono-6,3-lactone	17.033	0.00	13.64	0.00	0.00
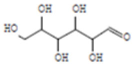	$C_6H_{12}O_6$	Glucose	17.867	0.00	0.10	0.00	0.00
	$C_{20}H_{42}O$	1-Eicosanol	18.133	0.00	0.00	0.00	0.04

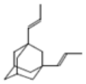	C16H24	1,3-Di(propen-1-yl)adamantane	18.142	0.00	0.00	0.32	0.00
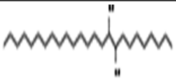	C25H50Br2	erythro-9,10-Dibromopentacosane	18.15	0.02	0.00	0.00	0.00
	C16H30O	Z,Z-8,10-Hexadecadien-1-ol	18.283	0.00	0.25	0.00	0.00
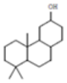	C17H30O	3-Phenanthrenol, tetradecahydro-4b,8,8-trimethyl-, [3S-(3.alpha.,4a.beta.,4b.alpha.,8a.beta.,10a.alpha.)]-	19.725	0.00	0.00	0.00	0.07
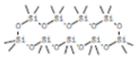	C18H54O9Si9	Cyclononasiloxane, octadecamethyl-	23.508	0.00	0.00	0.12	0.00
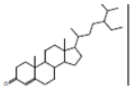	C29H48O	Stigmast-4-en-3-one	23.55	0.00	0.36	0.00	0.00
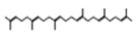	C30H50	2,6,10,14,18,22-Tetracosahexaene, 2,6,10,15,19,23-hexamethyl-, (all-E)-	24.058	0.00	0.25	0.00	0.00
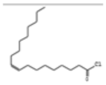	C18H33ClO	9-Octadecenoyl chloride	24.467	0.21	0.00	0.00	0.00
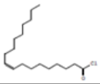	C18H33ClO	Oleoyl chloride	24.467	0.00	0.56	0.00	0.72
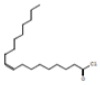	C18H33ClO	Oleoyl chloride	24.467	0.00	0.00	0.00	0.00
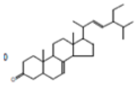	C29H46O	Spinasterone	24.892	0.00	0.65	0.00	0.00
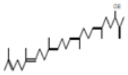	C30H50O	1,6,10,14,18,22-Tetracosahexaen-3-ol, 2,6,10,15,19,23-hexamethyl-, (all-E)-	25.342	0.00	0.16	0.00	0.00
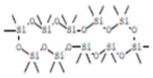	C20H60O10Si10	Cyclodecasiloxane, eicosamethyl-	26.208	0.00	0.00	0.00	0.10
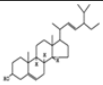	C29H48O	Stigmasta-5,22-dien-3-ol, (3.beta.,22E)-	29.017	0.12	0.64	0.00	0.28
		TOTAL		2.26	32.56	3.56	1.97
						40.35	

Table S2. GC/MS analysis of vertical compound change in brown part of agarwood

Sesquiterpenes

Compound name	RT	L1	L2	L3	L4	L5	L6
3,7-Cyclodecadiene-1-methanol, .alpha.,.alpha.,4,8-tetramethyl-, [s-(Z,Z)] $$ Hedycaryol	11.092	0.00	0.00	0.00	0.00	0.09	0.00
Lanceol, cis	11.425	0.05	0.06	0.05	0.00	0.29	0.00
1H-Cycloprop[e]azulene, decahydro-1,1,7-trimethyl-4-methylene-, [1aR-(1a.alpha.,4a.alpha.,7.alpha.,7a.beta.,7b.alpha.)	12.092	0.00	0.00	0.00	0.00	0.36	0.49
Azulene, 1,2,3,4,5,6,7,8-octahydro-1,4-dimethyl-7-(1-methylethylidene)-, (1S-cis)-	12.1	0.10	0.14	0.05	0.06	0.54	0.51
2-Naphthalenemethanol, decahydro-.alpha.,.alpha.,4a-trimethyl-8-methylene-, [2R-(2.alpha.,4a.alpha.,8a.beta.)]-	12.267	0.25	0.39	0.25	0.32	0.70	0.72
Humulene	12.875	0.10	0.12	0.09	0.12	0.30	0.30
(1R,4aR,7R,8aR)-7-(2-Hydroxypropan-2-yl)-1,4a-dimethyldecahydronaphthalen-1-ol	13.858	0.12	0.13	0.11	0.10	0.22	0.30
Tricyclo[4.4.0.0(2,7)]dec-8-ene-3-methanol, .alpha.,.alpha.,6,8-tetramethyl-, stereoisomer	15.208	0.11	0.00	0.00	0.12	0.22	0.28
Cyclohexanemethanol, 4-ethenyl-.alpha.,.alpha.,4-trimethyl-3-(1-methylethenyl)-, [1R-(1.alpha.,3.alpha.,4.beta.)]-	15.742	0.15	0.33	0.25	0.22	0.48	0.56
6-(1-Hydroxymethylvinyl)-4,8a-dimethyl-3,5,6,7,8,8a-hexahydro-1H-naphthalen-2-one	16.433	0.00	0.00	0.00	0.00	0.00	0.00
epi-.alpha.-Patchoulene	17.108	0.00	0.00	0.00	0.00	0.68	0.73
Germacra-1(10),4,11(13)-trien-12-oic acid, 6.alpha.-hydroxy-, .gamma.-lactone, (E,E)-	17.567	0.00	0.11	0.12	0.11	0.00	0.00
6-(1-Hydroxymethylvinyl)-4,8a-dimethyl-3,5,6,7,8,8a-hexahydro-1H-naphthalen-2-one	18.133	0.00	0.00	0.00	0.30	0.35	0.00
2-Iodophenyl-.beta.-phenylpropionate	20.425	0.17	0.24	0.00	0.00	0.00	0.00
TOTAL		1.03	1.52	0.92	1.36	4.24	3.88
					14.48		

Chromones

Compound name	RT	L1	L2	L3	L4	L5	L6
4H-1-Benzopyran-4-one, 2-methyl-	10.9	0.06	0.07	0.06	0.09	0.13	0.00
6-Methoxy-3-methyl-2-benzofurancarbaldehyde	20.742	0.00	0.00	0.00	0.00	0.00	0.00
2-Hydroxy-4-methoxy-7-methyl-7,8,9,10,11,12,13,14-octahydro-6-oxabenzocyclododecen-5-one	22.817	0.00	0.05	0.00	0.00	0.00	0.32
7H-Furo[3,2-g][1]benzopyran-7-one, 9-[(3-methyl-2-butenyl)oxy]-	23.483	0.00	0.00	0.00	0.00	0.00	0.00
6-Methoxy-2-phenethyl-4H-chromen-4-one	24.017	0.00	0.00	1.53	1.51	1.98	1.27
Coumarin, 6-benzyloxy-3,4-dihydro-4,4-dimethyl-	24.375	2.68	1.90	0.42	3.40	1.29	1.00
4H-1-Benzopyran-4-one, 5-hydroxy-7-methoxy-2-(4-methoxyphenyl)-	26.35	0.00	0.00	0.00	0.00	0.00	0.00
TOTAL		2.74	2.03	2.02	5.00	3.40	2.59
					19.89		

Alkaloids

Compound name	RT	L1	L2	L3	L4	L5	L6
DL-Proline, 5-oxo-, methyl ester	9.333	0.00	0.00	0.00	0.00	0.00	0.00
2,5-Dimethoxy-4-ethylamphetamine	11.158	0.00	0.00	0.00	0.05	0.10	0.00
beta.-D-glucosyloxyazoxymethane	17.283	0.00	0.00	0.00	0.00	1.83	1.79
Oxalic acid, monoamide monohydrazide, N-benzyl-N"-(1-oxo-3-phenylprop-2-enyl)-	17.667	0.00	0.00	0.00	0.00	0.00	0.44
Pentanal, 2-[bis(phenylmethyl)amino]-4-methyl-	23.558	3.45	0.42	0.38	0.00	0.00	0.00
Capsaicin	23.575	0.00	0.00	0.00	0.00	0.00	0.00
Nonivamide	23.75	0.00	0.00	0.00	0.00	0.00	0.00
2,6-Lutidine-4-[benzylamino]-3,5-dichloro	23.967	2.59	1.73	0.00	0.00	0.00	0.74
benzene-1,2-diol, 4-[2-nitroethenyl]-, 1-O-benzyl(ether)	24.225	0.00	0.00	0.00	0.00	0.00	0.64
Benzenepropanal, .alpha.-[bis(phenylmethyl)amino]-	25.675	1.23	0.00	0.00	0.00	0.00	0.00
TOTAL		7.27	2.15	0.38	0.05	1.93	3.60
					17.21		

Aromatic compounds

Compound name	RT	L1	L2	L3	L4	L5	L6
Hexadecanoic acid, methyl ester	16.017	0.14	0.21	0.19	0.34	0.55	0.70
n-Hexadecanoic acid	16.575	0.00	0.00	0.00	0.00	1.33	1.58
Ethyl 5,8,11,14,17-icosapentaenoate	17.925	0.23	0.00	0.19	0.29	0.20	0.57
9,12-Octadecadienoic acid (Z,Z)-, methyl ester	18.283	0.00	0.00	0.24	0.28	0.00	0.80
11-Octadecenoic acid, methyl ester	18.342	0.29	0.34	0.31	0.50	1.02	1.20
Octadecanoic acid, methyl ester	18.6	0.00	0.00	0.05	0.08	0.00	0.00
Oleic Acid	18.767	0.00	0.00	0.00	0.00	0.39	0.43
Hexadecanoic acid, 2-hydroxy-1-(hydroxymethyl)ethyl ester	23.067	0.05	0.00	0.08	0.11	0.00	0.18
9-Octadecenoic acid (Z)-, 2,3-dihydroxypropyl ester	24.475	0.00	0.58	0.00	0.58	0.00	0.00
TOTAL		0.71	1.14	1.07	2.19	3.49	5.47
					15.72		

Aromatic compounds

Compound name	RT	L1	L2	L3	L4	L5	L6
Benzaldehyde	4.592	0.22	0.31	0.30	0.28	0.51	0.70
Hydrocinnamic acid	8.908	0.00	0.11	0.00	0.00	0.41	0.48
2-Methoxy-4-vinylphenol	8.767	0.00	0.00	0.00	0.00	0.00	0.00
Phenol, 2,6-dimethoxy-	9.125	0.00	0.00	0.00	0.00	0.00	0.00
2-Butanone, 4-(4-methoxyphenyl)-	10.558	0.05	0.08	0.05	0.06	0.15	0.21
Phenol, 2,4-bis(1,1-dimethylethyl)-	10.617	0.09	0.00	0.00	0.08	0.12	0.19
2,4-Di-tert-butylphenol	10.625	0.00	0.07	0.07	0.00	0.00	0.00
Benzenepropanoic acid, 4-methoxy-, methyl ester	10.808	0.04	0.00	0.00	0.00	0.00	0.00
3-tert-Butyl-4-hydroxyanisole, acetate	11.158	0.00	0.00	0.06	0.00	0.00	0.00
Phenol, 3,4,5-trimethoxy-	11.5	0.00	0.00	0.00	0.00	0.00	0.00
Benzaldehyde, 4-hydroxy-3,5-dimethoxy-	12.167	0.00	0.00	0.00	0.00	0.00	0.00
Benzenepropanoic acid, 3,5-bis(1,1-dimethylethyl)-4-hydroxy-, methyl ester	16.442	0.00	0.00	0.00	0.00	0.00	0.00
Naphthalene, 1,2,3,4-tetrahydro-1-nonyl-	17.642	0.00	0.00	0.00	0.00	0.37	0.00
1-Penten-3-one, 1,5-diphenyl-	19.292	0.17	0.00	0.31	0.23	0.79	0.00
2-Iodophenyl-.beta.-phenylpropionate	20.417	0.00	0.00	0.00	0.28	0.00	0.00
3-Pentanone, 1,5-diphenyl-	20.433	0.00	0.00	0.34	0.00	0.63	0.69
8-Naphthol, 1-(benzyloxy)-	21.558	0.00	0.00	0.00	0.00	0.00	1.01
1,2-Benzenedicarboxylic acid, diisooctyl ester	23.425	0.00	0.00	0.00	0.00	0.00	0.34
TOTAL		0.56	0.57	1.12	0.93	2.97	3.62
						10.92	

Terpenoids

Compound name	RT	L1	L2	L3	L4	L5	L6
2-Butanone, 4-phenyl-	8	0.29	0.49	0.40	0.37	0.83	1.13
Benzenepropanoic acid, methyl ester	8.333	0.06	0.09	0.06	0.07	0.16	0.16
Benzylmalonic acid	8.9	0.07	0.00	0.00	0.10	0.00	0.00
3-(4-Methoxyphenyl)propionic acid	11.258	0.00	0.00	0.00	0.00	0.15	0.18
4-((1E)-3-Hydroxy-1-propenyl)-2-methoxyphenol	13.117	0.27	0.25	0.15	0.26	0.52	0.80
Androsta-1,4-dien-3-one, 17-hydroxy-17-methyl-, (17.alpha.)-	14.3	0.00	0.00	0.00	0.00	0.24	0.23
1H-2,8a-Methanocyclopenta[a]cyclopropa[e]cyclodecen-11-one, 1a,2,5,5a,6,9,10,10a-octahydro-5,5a,6-trihydroxy-1,4-	16.975	0.00	0.00	0.00	0.00	0.00	0.96
TOTAL		0.69	0.83	0.60	0.80	1.89	3.46
					9.25		

Compound name	RT	L1	L2	L3	L4	L5	L6
2-Propanamine, N-methyl-N-nitroso-	6.708	0.00	0.00	0.00	0.00	0.00	0.00
4H-Pyran-4-one, 2,3-dihydro-3,5-dihydroxy-6-methyl-	6.858	0.00	0.00	0.00	0.00	0.00	0.00
3-Hydroxy-2-methylglutaric acid dimethyl ester	7.717	0.23	0.22	0.12	0.36	0.69	0.59
2-Pentene, 2,4,4-trimethyl-	9.2	0.00	0.00	0.00	0.00	0.00	0.00
1,3-Propanediol, 2-(hydroxymethyl)-2-nitro-	10.117	0.00	0.00	0.00	0.00	0.00	0.00
D-Allose	10.367	0.10	0.11	0.08	0.13	0.00	0.00
7-Nonenamide	10.517	0.00	0.00	0.00	0.00	0.00	0.00
Santalol	11.417	0.00	0.00	0.00	0.06	0.00	0.00
alpha.-Farnesene	11.433	0.00	0.00	0.00	0.00	0.00	0.29
1,2,3,5-Cyclohexanetetrol, (1.alpha.,2.beta.,3.alpha.,5.beta.)-	11.667	0.00	0.00	0.00	0.00	0.00	0.00
Cyclooctasiloxane, hexadecamethyl	12.075	0.00	0.00	0.00	0.00	0.00	0.00
1-(3-Methyl-cyclopent-2-enyl)-cyclohexene	15.217	0.00	0.13	0.12	0.00	0.00	0.00

Others
